# Isolation of *Coxiella burnetii* in patients with nonspecific febrile illness in South Korea

**DOI:** 10.1186/s12879-020-05130-3

**Published:** 2020-06-17

**Authors:** Seung Hun Lee, Jae Hoon Lee, Sungdo Park, Hae Kyung Lee, Seon Do Hwang, Hye Won Jeong, Jung Yeon Heo, Yeong Seon Lee

**Affiliations:** 1 Division of Bacterial Disease Research, Center for Infectious Disease Research, Korea National Institute of Health, Osongsaengmyeong 2-ro, Cheongju-si, 28159 South Korea; 2 Yeosu National Quarantine station, Yeosu-si, Jeollanam-do 58729 South Korea; 3 Wonkwang University College of Medicine, Iksan-si, Jeollabuk-do 54538 South Korea; 4 Chungbuk National University College of Medicine, Cheongju-si, Chungcheongbuk-do 28644 South Korea; 5 Division of Infectious Diseases, Ajou University School of Medicine, Suwon-si, Kyeonggi-do 16499 South Korea

**Keywords:** *Coxiella burnetii*, Q fever, Culture isolation, molecular sequencing, electron microscopy

## Abstract

**Background:**

The number of human Q fever cases in South Korea has been rapidly increasing since 2015. We report the first isolation of *Coxiella burnetii* in Korea in two patients who initially presented with non-specific febrile illness and were finally diagnosed with acute Q fever in South Korea.

**Case presentation:**

Two adult patients with fever had serologic tests against *C. burnetii* initially negative, and polymerase chain reaction against 16S rRNA using whole blood was also negative. After bacterial amplification of *C. burnetii* in immune-depressed mice, we isolated *C. burnetii* from patients with acute Q fever. The isolates KZQ2 and KZQ3 were confirmed by polymerase chain reaction, nucleotide sequence analysis, and morphologic observation using a transmission electron microscope.

**Conclusions:**

These results can help us understand the clinical and epidemiologic features of Q fever in South Korea.

## Background

Acute Q fever is an infectious zoonotic disease that is characterized by a sudden onset of fever, headache, and atypical pneumonia. It is primarily diagnosed via a serologic test, and paired samples from the acute and convalescent phases taken several weeks apart are required to confirm the diagnosis. Because of these nonspecific symptoms and diagnostic challenges, acute Q fever has been considered an under-recognized and underdiagnosed infectious disease, particularly in non-endemic or non-epidemic areas. Although acute Q fever is generally self-limiting and resolves in < 14 days, even in untreated patients, it can lead to public health concerns, such as the large-scale outbreaks in the Netherlands, and has the potential of being used as a bioterrorism agent [1, 2]. Furthermore, chronic Q fever can develop after symptomatic or asymptomatic acute infection; for example, as endocarditis or vascular infection. Chronic Q fever develops in approximately < 5% of persons with acute infection [3]. It is therefore necessary to understand the microbiologic and molecular characteristics of *Coxiella burnetii* in patients with acute Q fever from different geographic areas. However, it is usually not possible to isolate *C. burnetii* from acutely infected patients because of the difficulty in obtaining suitable samples or handling them safely. Nevertheless, to control diseases and understand epidemiologic features in during a Q fever epidemic, we should understand the microbiologic characteristics of circulating *C. burnetii* through isolation.

A rapid increase in the annual incidence of Q fever has recently occurred in South Korea, with 0.16 cases per 100,000 persons in 2016 and 0.19 cases per 100,000 persons in 2017. Compared to 0.02 cases per 100,000 persons in 2008, this represents a greater than eight-fold increase. The Korea Centers for Disease Control and Prevention reported that the number of confirmed cases of Q fever was 8 in 2014, 27 in 2015, 81 in 2016, 96 in 2017, and 163 in 2018. Although the number of confirmed Q fever cases has been increasing, there have been no cases on the isolation of *C. burnetii* from human blood in Korea [4]. Here, we report the isolation of *C. burnetii* in two patients who initially presented with non-specific febrile illness but were finally diagnosed with acute Q fever.

## Case presentation

Patient 1, a 32-year-old man office worker living in the outskirts of Cheongju-si, Chungcheongbuk-do, South Korea, was admitted to a hospital with a 5-day history of fever and headache in March 2016. On physical examination, he had no remarkable findings except for a body temperature of 39.6 °C. Laboratory tests showed normal platelet (217 × 10^3^/μL) and white blood cell (5720/μL) counts with elevated C-reactive protein (8.27 mg/dL), aspartate aminotransferase (71 IU/L), and alanine transaminase (76 IU/L) levels. Although intravenous ceftriaxone was initiated as an empiric antibiotic treatment for the febrile illness, no bacterial or fungal organisms were isolated in the blood samples. Because of the persistent fever (> 7 days) despite antibiotic treatment, a serum sample was collected for specific *C. burnetii* antibody and nucleic acid detection on hospital day 4. The patient had no history of animal contact. Although ceftriaxone was only administered for 5 days, the patient was discharged in an afebrile state after 9 days in the hospital.

Patient 2, a previously healthy 65-year-old man, visited an outpatient clinic in May 2016 with a 1-month history of fever and general weakness. He lived in Buan-gun, Cheollabuk-do, South Korea and worked as a dairy cattle raiser. Physical examination revealed an elevated body temperature of 38.5 °C but no other remarkable findings. A complete blood count showed mild thrombocytopenia (platelet count, 142 × 10^3^/μL) and a normal white blood cell count (4050/μL) with 59.8% neutrophils. Blood biochemistry revealed elevated C-reactive protein (3.11 mg/dL), aspartate aminotransferase (44 IU/L), and alanine transaminase (40 IU/L) levels. Given his history of animal contact, a blood sample for *C. burnetii* antibody testing and isolation was collected before administering oral doxycycline as an empirical antibiotic treatment. After doxycycline was prescribed for 7 days, his symptoms gradually improved.

We used an indirect fluorescent antibody (IFA) assay from a commercial kit (IF0200G, IF0200M, Focus Diagnostics, Cyprus, California, USA). The initial serum samples, which were obtained on about days 9 and 30 of symptom onset, were negative for phase II IgG and IgM against *C. burnetii*. Furthermore, the phase I IgG and IgM antibodies were negative. To detect the gene of the causative antigen, we performed nested polymerase chain reaction (PCR) assays on blood samples of the patients and primers specific to the 16S rRNA of *Anaplasma phagocytophilum, Ehrlichia chaffeensis, C. burnetii,* and the outer membrane protein (*omp*) of *Rickettsia spp.* All PCR test results were negative; however, after 9 and 14 weeks, for patients 1 and 2 the phase II IgG and IgM titers were ≥ 1:2048 and 1:16, respectively, from serum samples in the convalescence phase. The phase I IgG and IgM titers were 1:512 and 1:64 for patient 1 and 1:512 and 1:128 for patient 2.

To amplify the bacteria, we inoculated 400 μL buffy coats from the patients’ blood into immune-depressed BALB/c mice via the intraperitoneal route. On day 49 post-infection (dpi 49), the spleens of the infected animals that had developed splenomegaly were harvested (Additional file 1: Figure S1). The spleens were homogenized for cell culture and gene identification, and DNA was extracted for PCR analyses of the specific genes described above. The PCR results were positive for 16S rRNA, *IS1111,* and *omp*-specific genes of *C. burnetii*.

**Fig. 1:**
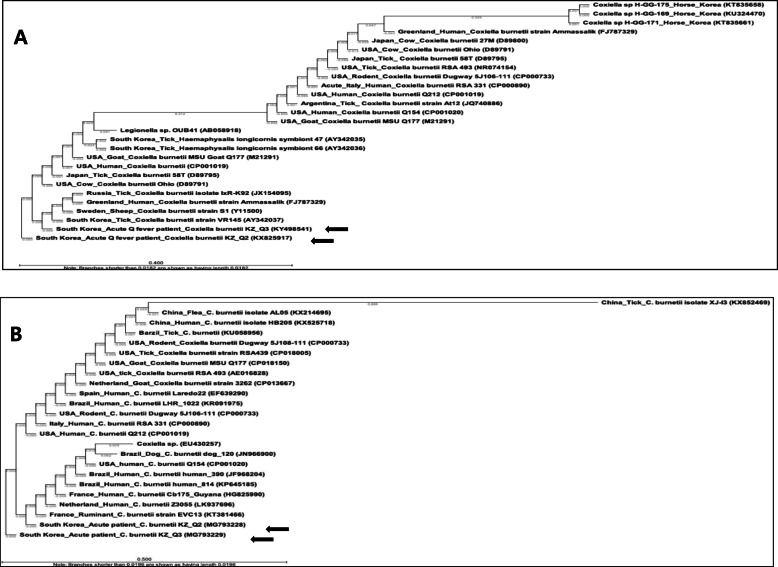
Partial sequences of the 16S rRNA and *IS1111* of *Coxiella burnetii* isolates obtained from two patients with Q fever in South Korea (arrows). **a** 16S rRNA and **b***IS1111*. The phylogenetic trees were constructed using the Jukes-Cantor/Neighbor joining methods. The locations, hosts, and GenBank accession numbers are indicated. The branch lengths of the trees show the evolutionary distances, and the numbers on the branches indicate bootstrap support (1000 replicates)

The nucleotide sequences of the isolates KZQ2 (patient 1) and KZQ3 (patient 2) [16S rRNA (KX825917, KY498541), *IS1111* (MG793228, MG793229)*,* and *omp* (MG836249, MG836250)] were matched to *C. burnetii*. Comparison of the 16S rRNA nucleotide sequences showed high similarity (99.6–100%) with *C. burnetii* sequences (USA: CP001019, USA: D89791, Japan: D89795) (Fig. 1a). The sequences were identical to South Korea: KX825917, KY498541, and AY342037 as well as USA: M21291. The nucleotide sequences of *IS1111* were identical to those of *C. burnetii* (Fig. 1b); those of *omp* showed 80.30–99.75% sequence similarity to tick and human sequences from South Korean isolates (tick: AY342038, human: KM11542, MG836249, and MG836250).

Homogenized spleens were infected in the African green monkey kidney epithelial cell line Vero (KCLB-10081) in RPMI1640 medium (GIBCO, Waltham) supplemented with 2% fetal bovine serum (GIBCO, Waltham) and 0.4 mM L-glutamine (GIBCO, Waltham) and cultured in an incubator at 37 °C with 5% CO_2_; the medium was changed regularly. Before cell passage, the infected cells were examined under a transmission electron microscope after Diff-Quik (Sysmex, Japan) staining and in-house IFA testing.

Bacterial isolation from the animals was performed under Animal Biosafety Level-3 conditions, and cell infections were performed under Biosafety Level 3 conditions. All protocols using live animals were reviewed and approved by an Institutional Animal Care and Use Committee (IACUC, KCDC-045-16-2A) and we used for four-week-old Balb/c mice were purchased from Orientbio (Seoul, South Korea) which used CO2 euthanasia.

**Fig. 2:**
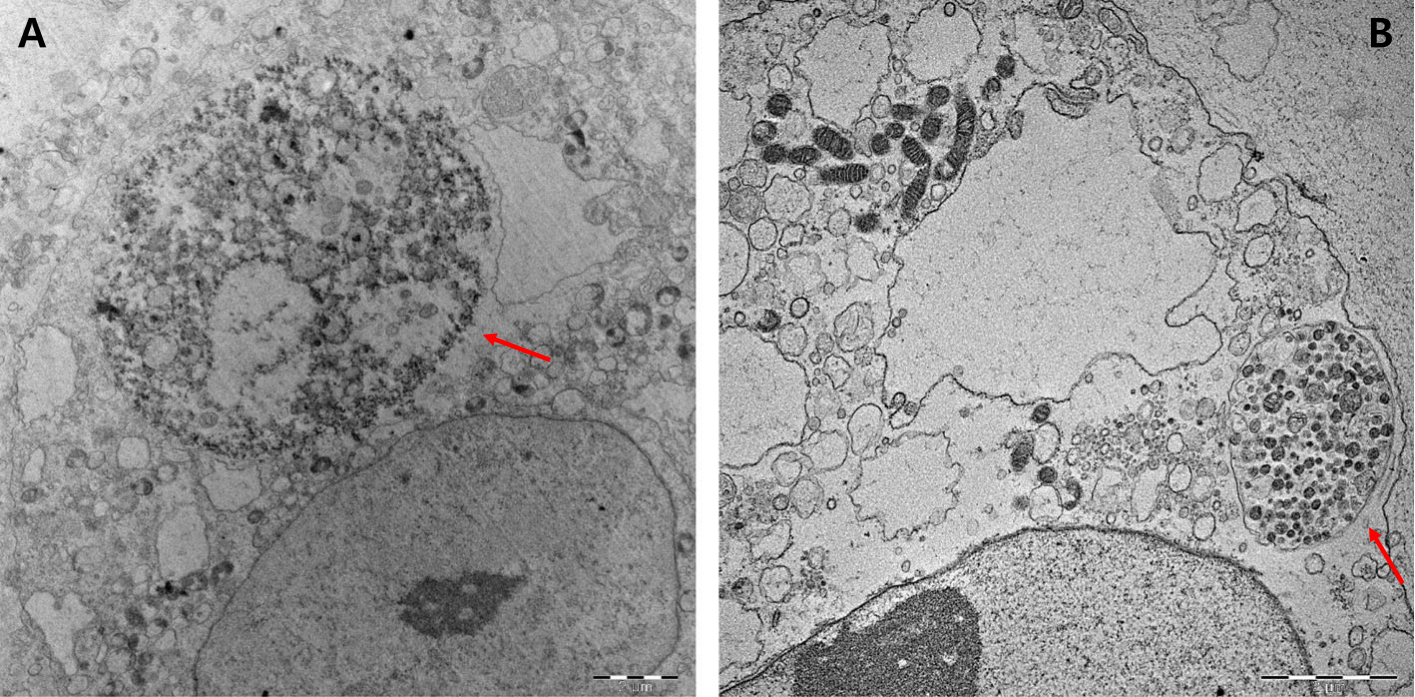
Morphology of the isolated *Coxiella burnetii* in Vero cells visualized by transmission electron microscopy. Vero cell monolayers were incubated with homogenates of *C. burnetii*-infected mice spleen, and fresh medium was added. The infected cells were fixated and processed for transmission electron microscopy on post-infection day 60. Bar, 2 μm. The arrow shows the replicated *C. burnetii* (**a**: KZQ2, **b**: KZQ3)

During cell infection, *C. burnetii* antigens were confirmed with the in-house IFA method using seropositive serum (Focus Diagnostics, CA) as the primary antibody and human IgG-fluorescein-conjugated isothiocyanate (Focus Diagnostics, CA) as the secondary antibody. Every 2 weeks, the infected cells were passaged. On dpi 3, we observed fluorescent *C. burnetii* antigens in the cell pellet using the in-house IFA test (Additional file 2: Figure S2). On dpi 10, an evaluation of the morphology of *C. burnetii* with Diff-Quik (Sysmex, Japan) staining (Additional file 3: Figure S3) and transmission electron microscopy revealed replication of *C. burnetii* in the vacuole within the cytoplasm of the infected cells (Fig. 2).

## Discussion and conclusions

We first isolated *C. burnetii* from the whole blood of two patients with acute Q fever who presented with non-specific febrile illness in South Korea. Although the IFA against *C. burnetii* and PCR of 16S rRNA were initially negative, we were able to isolate *C. burnetii* after bacterial amplification in immune-depressed mice. The *C. burnetii* isolates KZQ2 and KZQ3 were verified by PCR, sequence analysis, and morphologic observations.

The molecular characteristics of *C. burnetii* have been previously reported from milk samples, and *C. burnetii* was detected in ticks, livestock, and humans in South Korea [5–9]. We assume that *C. burnetii* may be more widespread in South Korea than previously reported. To confirm this suspicion, additional investigations in patients, animals, ticks, and environmental samples are needed.

It is difficult to identify patients with acute Q fever if they initially have negative serologies against *C. burnetii*. To accurately confirm acute Q fever, serologic tests should be conducted using paired sera from the acute and convalescent stages taken 4–6 weeks apart. However, symptomatic acute Q fever usually presents the duration of fever within 2 weeks [10, 11]. Without epidemiologic factors such as livestock raiser and slaughterhouse worker, patients with acute Q fever who have negative serology at the acute stage of illness are more likely to be neglected. This is the reason two cases in our study were confirmed to have acute Q fever at 9 and 14 weeks after the initial serologic test. Delayed diagnosis also hinders proper monitoring of patients with chronic Q fever. Most physicians usually consider that bacterial infection does not progress to chronic infections. Therefore, no follow up was organized for these patients, and we could not perform echocardiography and monitor their follow-up serology.

Although quantitative PCR could not be performed, we demonstrated bacterial isolation using methods such as TEM, Diff-Quik, IFA, and genetic analysis from cell culture. It may be meaningful that the study is useful because the extant clinical isolate has been obtained from Korean Q fever patient, which will provide a basis for characterizing a human isolate and identifying isolate methods in South Korea.

The human isolates KZQ2 and KZQ3 identified in the present case study are the first isolates from patients with Q fever in South Korea. These results provide a clue to understanding the bacterial characterization of *C. burnetii* isolated from Korean patients and might help healthcare providers understand the clinical and epidemiologic features of these patients. Future studies should analyze the pathogenicity and infectivity of Korean *C. burnetii* isolates.

## Supplementary information

**Additional file 1: Figure S1.** The spleen of a Balb/c mouse infected with the patient’s buffy coat; day 49; splenomegaly. The spleen is twice as large as the normal.

**Additional file 2: Figure S2.** Light micrograph of *Coxiella burnetii* cultured in African green monkey kidney epithelial cell line (Vero) (KZQ2 and KZQ3; Diff-Quik staining). Original magnification (A and B; × 1000).

**Additional file 3: Figure S3.** In-house immunofluorescence staining of isolate *Coxiella burnetii* from patient in African green monkey kidney epithelial cell line (Vero) (dpi 3). Culture preparations stained by IFA using an anti-*C. burnetii* serum. Green indicates intracytoplasmic inclusions filled with numerous bacteria. Fluorescence magnification (A-B; × 400).

## Data Availability

The data used and/or analyzed during the current study are available from the corresponding author on reasonable request.

## Abbreviations

*C. burnetii*: *Coxiella burnetii*
IFA: Indirect fluorescent antibody
PCR: Polymerase chain reaction
omp: outer membrane protein
