# Supplementary figures and images for: Isolation of Coxiella burnetii in patients with nonspecific febrile illness in South Korea
Source: BMC Infect Dis. 2020 Jun 17;20:421. doi: 10.1186/s12879-020-05130-3 (PMC7301556; doi:10.1186/s12879-020-05130-3)

## Slide 1
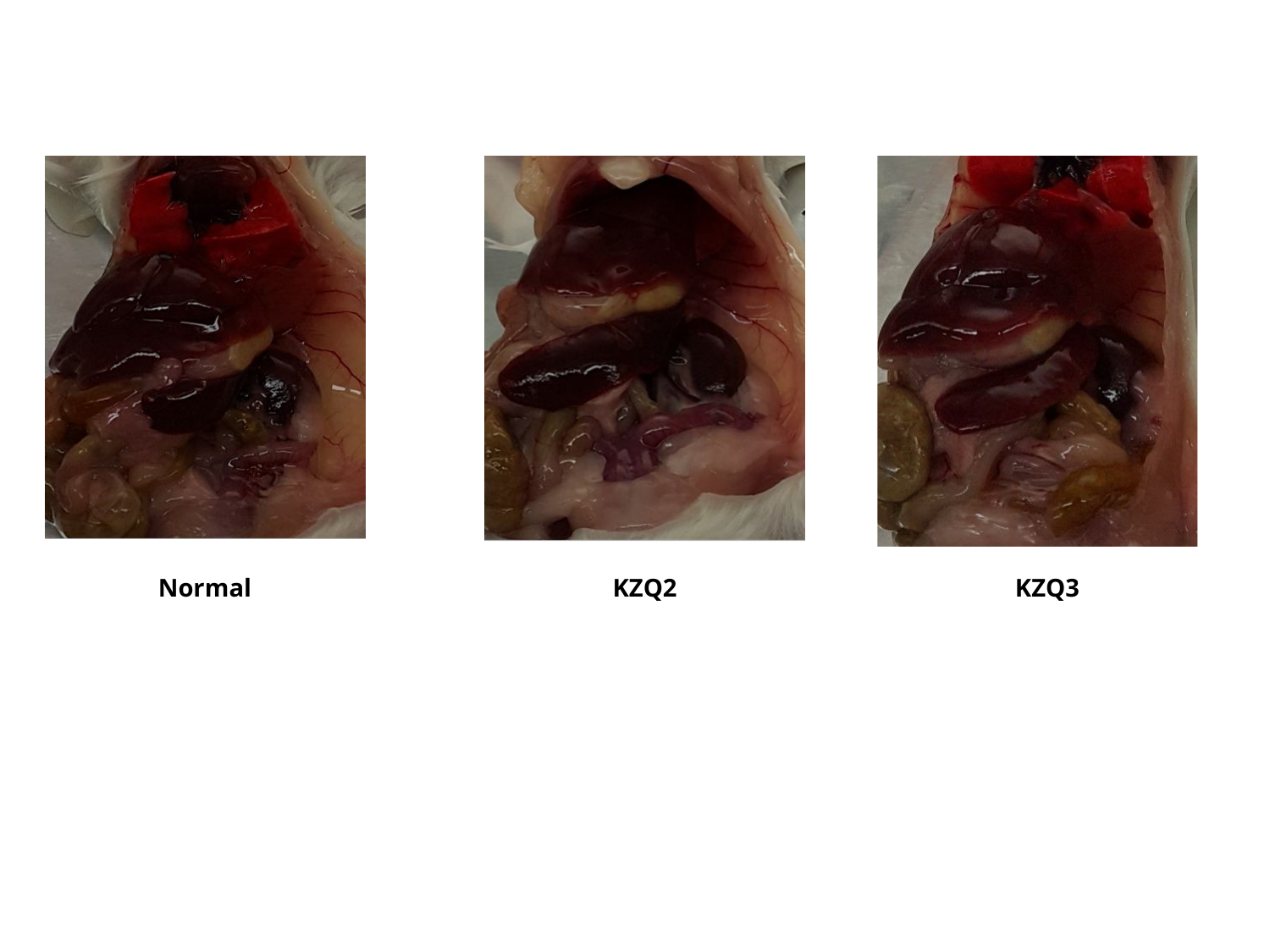

Normal
KZQ2
KZQ3

Supplement: Supplementary file 1 — Additional file 1: Figure S1. The spleen of a Balb/c mouse infected with the patient’s buffy coat; day 49; splenomegaly. The spleen is twice as large as the normal. [file 12879_2020_5130_MOESM1_ESM.pptx]

## Slide 1
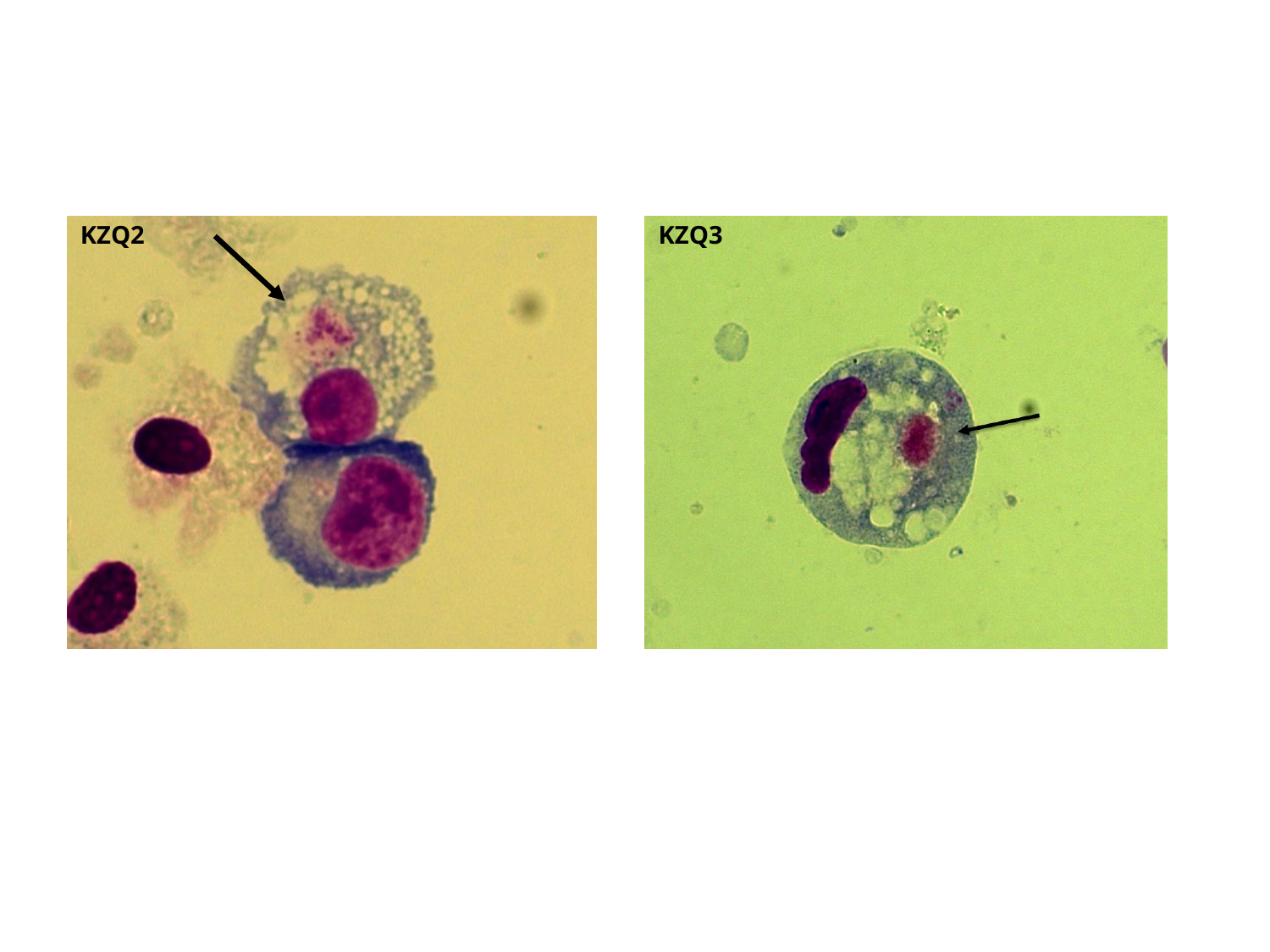

KZQ3
KZQ2

Supplement: Supplementary file 2 — Additional file 2: Figure S2. Light micrograph of Coxiella burnetii cultured in African green monkey kidney epithelial cell line (Vero) (KZQ2 and KZQ3; Diff-Quik staining). Original magnification (A and B; × 1000). [file 12879_2020_5130_MOESM2_ESM.pptx]

## Slide 1
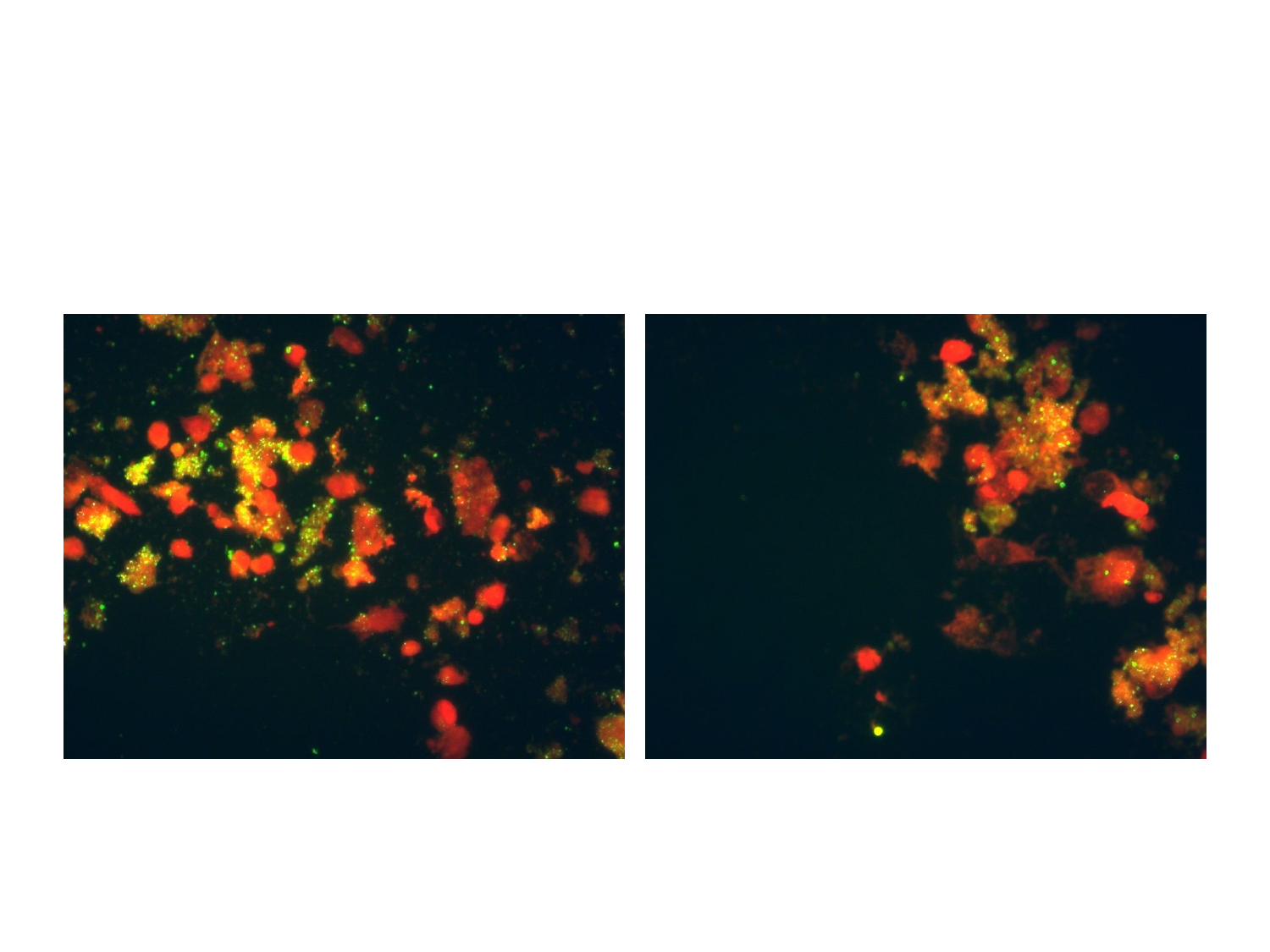

Supplement: Supplementary file 3 — Additional file 3: Figure S3. In-house immunofluorescence staining of isolate Coxiella burnetii from patient in African green monkey kidney epithelial cell line (Vero) (dpi 3). Culture preparations stained by IFA using an anti-C. burnetii serum. Green indicates intracytoplasmic inclusions filled with numerous bacteria. Fluorescence magnification (A-B; × 400). [file 12879_2020_5130_MOESM3_ESM.pptx]
